# Discovery and validation of PZP as a novel serum biomarker for screening lung adenocarcinoma in type 2 diabetes mellitus patients

**DOI:** 10.1186/s12935-021-01861-8

**Published:** 2021-03-10

**Authors:** Jiayue Yang, Cheng Yang, Hong Shen, Wenjun Wu, Zhen Tian, Qinghua Xu, Cuiping Cao, Shugao Ye, Le Ban, Xin Tong, Jie Mei

**Affiliations:** 1 Department of Endocrinology, Wuxi People’s Hospital Affiliated to Nanjing Medical University, Wuxi, 214023 China; 2 Department of Gastroenterology, Wuxi People’s Hospital Affiliated to Nanjing Medical University, Wuxi, 214023 China; 3 Department of Clinical Laboratory, Wuxi People’s Hospital Affiliated to Nanjing Medical University, Wuxi, 214023 China; 4 Department of Chest Surgery, Wuxi People’s Hospital Affiliated to Nanjing Medical University, Wuxi, 214023 China; 5 Department of Oncology, Wuxi People’s Hospital Affiliated to Nanjing Medical University, No. 299 Qingyang Road, Wuxi, 214023 China

**Keywords:** Biomarker, Lung adenocarcinoma, Type 2 diabetes mellitus, Mass spectrum

## Abstract

**Background:**

Patients with type 2 diabetes mellitus (T2DM) have an increased risk of suffering from various malignancies. This study aimed to identify specific biomarkers that can detect lung adenocarcinoma (LAC) in T2DM patients for the early diagnosis of LAC.

**Methods:**

The clinical information of hospitalized T2DM patients diagnosed with various cancers was collected by reviewing medical records in Wuxi People’s Hospital Affiliated to Nanjing Medical University from January 1, 2015, to June 30, 2020. To discover diagnostic biomarkers for early-stage LAC in the T2DM population, 20 samples obtained from 5 healthy controls, 5 T2DM patients, 5 LAC patients and 5 T2DM patients with LAC (T2DM + LAC) were subjected to sequential windowed acquisition of all theoretical fragment ion mass spectrum (SWATH-MS) analysis to identify specific differentially-expressed proteins (DEPs) for LAC in patients with T2DM. Then, these results were validated by parallel reaction monitoring MS (PRM-MS) and ELISA analyses.

**Results:**

Lung cancer was the most common malignant tumor in patients with T2DM, and LAC accounted for the majority of cases. Using SWATH-MS analysis, we found 13 proteins to be unique in T2DM patients with early LAC. Two serum proteins were further validated by PRM-MS analysis, namely, pregnancy-zone protein (PZP) and insulin-like growth factor binding protein 3 (IGFBP3). Furthermore, the diagnostic values of these proteins were validated by ELISA, and PZP was validated as a novel serum biomarker for screening LAC in T2DM patients.

**Conclusions:**

Our findings indicated that PZP could be used as a novel serum biomarker for the identification of LAC in T2DM patients, which will enhance auxiliary diagnosis and assist in the selection of surgical treatment at an early stage.

**Supplementary Information:**

The online version contains supplementary material available at 10.1186/s12935-021-01861-8.

## Background

Diabetes mellitus is a group of metabolic disorders characterized by chronic hyperglycemia caused by complicated etiologies. Statistical data organized by the International Diabetes Federation revealed that there were approximately 387 million people worldwide who had diabetes mellitus in 2014, which is estimated to increase to 592 million in 2035 [1]. Diabetes mellitus occurs when the body cannot produce enough insulin or use insulin effectively. The former is defined as type 1 diabetes mellitus (T1DM), and the latter is type 2 diabetes mellitus (T2DM) [2]. Increasing evidence has revealed that T2DM is associated not only with microvascular complications (including nephropathy, retinopathy and neuropathy) and macrovascular complications (such as cardiovascular diseases) [3] but also with the oncogenesis and development of multiple types of cancer, including lung cancer, breast cancer and pancreatic cancer [4, 5].

Cancer is gradually becoming the first cause of mortality worldwide with growing numbers of estimated new cases and deaths each year [6]. Increasing evidence supports a direct association between T2DM and cancer with higher risks of cancer morbidity and mortality, especially for some of the most common malignancies [7]. To date, several mechanisms underlying the cancer-T2DM association have been explored, uncovering dysregulations of the insulin-like growth factor (IGF) system as the most important paradigm [7, 8]. However, despite the higher risk of cancer morbidity in the T2DM population, reliable biomarkers for screening and early diagnosis of specific types of cancer in T2DM patients have not yet been discovered.

Mass spectrum (MS)-dependent strategies offer novel insights for the identification and validation of disease-related biomarkers [9, 10]. For example, Geyer et al*.* developed a plasma proteome analysis pipeline using label-free quantitative MS, which detected 284 ± 5 proteins containing > 40 FDA-approved biomarkers without removing high-abundance proteins [11]. Sequential windowed acquisition of all theoretical fragment ion mass spectrum (SWATH-MS) is a newly developed strategy using a data-independent acquisition (DIA) method with high quantitative accuracy and reproducibility [12]. Using this strategy, increasing numbers of disease biomarkers have been identified, and novel criteria for disease typing based on proteomics have been established [13–15].

In this research, we first collected clinical information of hospitalized T2DM patients diagnosed with cancer and found that lung cancer was the most common malignant tumor in patients with T2DM in our cohort, with lung adenocarcinoma (LAC) accounting for the majority of cases. Using SWATH-MS and parallel reaction monitoring MS (PRM-MS) analyses, we discovered and preliminarily validated pregnancy zone protein (PZP) and insulin-like growth factor binding protein 3 (IGFBP3) as potential biomarkers. ELISA analysis was next used to further validate these biomarkers, and PZP was determined as a novel serum biomarker for screening LAC in T2DM patients, which will enhance auxiliary diagnosis and assist in the selection of early surgical therapeutics for LAC.

## Methods

### Patients and sample description

**Fig. 1:**
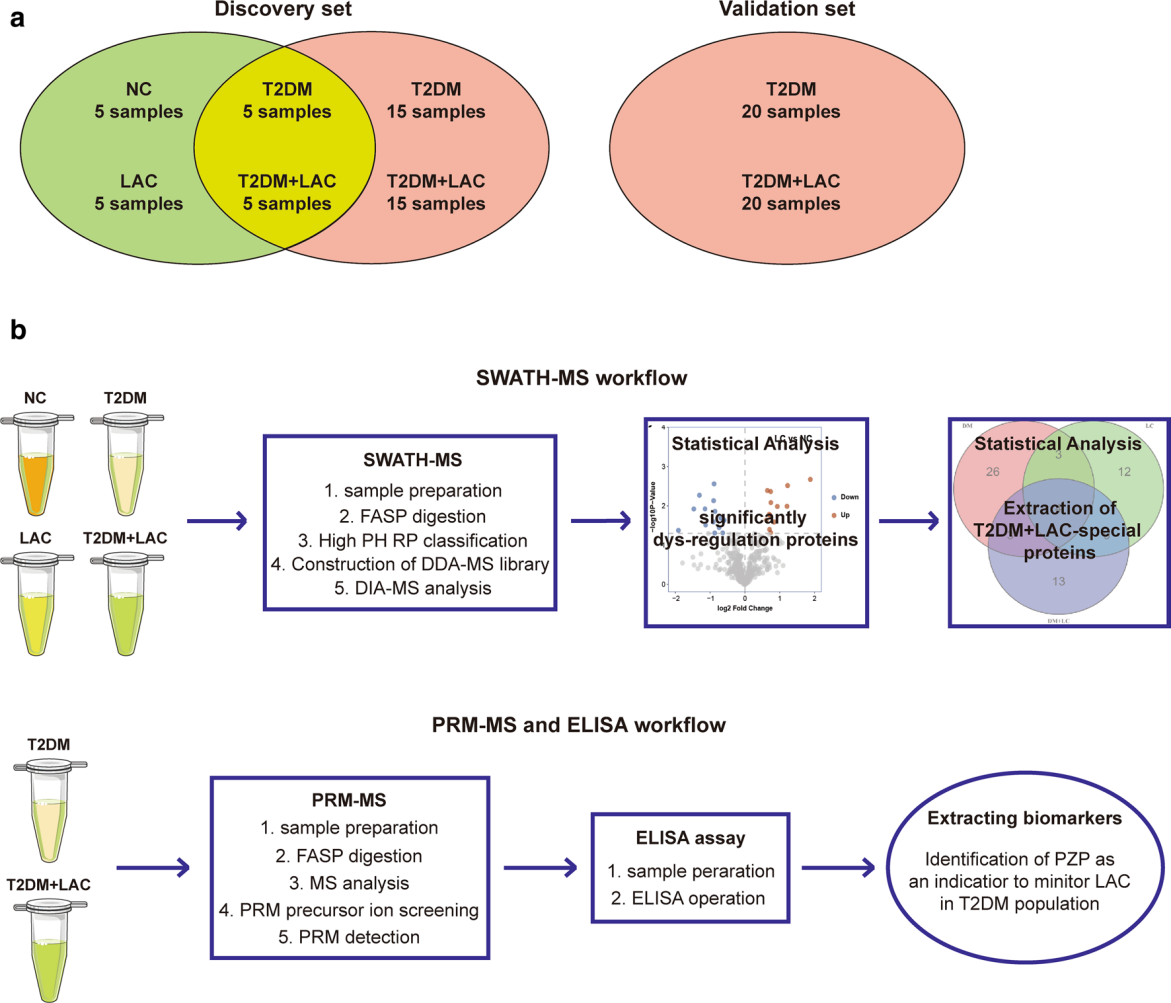
Overview of the study design. **a** Schematic diagram of serum specimens included in this study. **b** Schematic representation of the steps followed for the screening of the diagnostic proteins for LAC in T2DM patients (top) and the validated procedures (bottom)

The clinical information of hospitalized T2DM patients diagnosed with cancer was collected by reviewing medical records in Wuxi People’s Hospital Affiliated to Nanjing Medical University from January 1, 2015, to June 30, 2020. The following two cohorts were used to discover and validate biomarkers (Fig. 1a): In the discovery set, a total of 20 serum samples from 5 healthy controls, 5 T2DM patients, 5 LAC patients at TNM stage 1 and 5 T2DM patients with LAC at TNM stage 1 (T2DM + LAC), which were submitted to SWATH-MS analysis; besides, 20 serum samples from T2DM patients and 20 serum samples from T2DM patients with LAC at TNM stage 1 were submitted for PRM-MS and ELISA analysis. In the validation set, 20 serum samples from T2DM patients and 20 serum samples from T2DM patients with LAC at TNM stage 1 were collected for ELISA analysis. Before analysis, the serum samples were kept at −80 °C until use. The study was approved by the Ethical Committee at Wuxi People’s Hospital Affiliated to Nanjing Medical University, and the study was performed according to the Declaration of Helsinki.

### SWATH-MS analysis

#### Sample preparation

An Agilent Multiple Affinity Removal LC Column (Human 14) (Agilent, CA, USA) was used to remove high-abundance proteins in accordance with the protocol to obtain a low-abundance component solution in the serum sample. A 5 kD ultrafiltration tube was used for ultrafiltration and concentration, and one-fold volume of SDT lysis was added into the system, which was incubated in a water bath at 100 °C for 10 min and centrifuged at 14,000 × g for 15 min. The supernatant was extracted for protein quantification using a BCA kit, and the samples were subpackaged and stored at −80 °C.

#### FASP digestion

DTT was added to 200 μg of protein solution collected from each sample to reach a final concentration of 100 mM, and the samples were incubated in a water bath at 100 °C for 5 min. UA buffer (200 μL) was then added, and the samples were mixed and transferred to a 30 kD ultrafiltration centrifuge tube. The samples were centrifuged at 12,500 × g for 25 min, and the filtrate was discarded (this step was repeated twice). IAA buffer (100 μL; 100 mM IAA in UA) was then added, and the samples were shaken at 600 rpm for 1 min. The samples were allowed to react at room temperature for 30 min in the dark and then centrifuged at 12,500 × g for 25 min. UA buffer (100 μL) was then added, and the samples were centrifuged at 12,500 × g for 15 min (this step was repeated twice). Then, 40 mM NH_4_HCO_3_ (100 μL) was added, and the samples were centrifuged at 12,500 × g for 15 min (this step was repeated twice). Trypsin buffer (40 μL; 4 μg of trypsin in 40 μL of 40 mM NH_4_HCO_3_) was then added, and the samples were shaken at 600 rpm for 1 min and placed at 37 °C for 16–18 h. The collection tube was replaced, and the samples were centrifuged at 12,500×g for 15 min followed by the addition of 20 μL of 40 mM NH_4_HCO_3_ and centrifugation at 12,500×g for 15 min to collect the filtrate. A C18 cartridge was used to desalt the peptides. After the peptides were dried, they were reconstituted with 40 μL of 0.1% formic acid solution.

#### High PH RP classification

The peptide mixtures of all samples were submitted for fractionation using the Agilent 1260 infinity II HPLC system. Buffer A solution consisted of 10 mM HCOONH_4_ and 5% ACN (pH 10), and solution B consisted of 10 mM HCOONH_4_ and 85% ACN (pH 10). The chromatographic column was balanced with buffer A, and the sample was loaded by the autosampler onto the chromatographic column (XBridge Peptide BEH C18 Column, 130 Å, 5 µm, 4.6 mm × 100 mm; Waters, MA, USA) for separation with a flow rate of 1 mL/min. The liquid phase gradient was as follows: linear gradient of 5% B to 45% B within 40 min with a column temperature maintained at 30 °C. In total, 36 components were collected, and each component was dried in a vacuum concentrator for use. The sample was lyophilized, reconstituted with 0.1% formic acid aqueous solution and combined into 12 fractions.

#### Construction of DDA-MS library

From each fraction, 6 μL was removed and added to 2 μL of 10 × iRT standard peptide, and 2 μL of each sample was separated with nano-LC and analyzed by online electrospray tandem MS. The complete liquid-mass tandem system consisted of a liquid system (Waters Acquity UPLC; Waters, MA, USA) and an MS system (Q-Exactive HF; Thermo Fisher Scientific, MA, USA). Buffer A consisted of 0.1% formic acid aqueous solution, and buffer B consisted of 0.1% formic acid acetonitrile aqueous solution (acetonitrile was 80%). The sample was separated by an analytical column (Thermo Fisher Scientific, MA, USA; Acclaim PepMap C18, 75 μm × 25 cm) at a flow rate of 200 nL/min with the following gradient: 0–5 min, 1% B; 5–95 min, 1% B to 28% B; 95–110 min, 28% B to 38% B; 110–115 min, 38% B to 100% B; and 115–120 min, 100% B. The electrospray voltage was 2.0 kV. The MS parameters were set as follows: (1) MS: scan range (m/z) = 350–1600, resolution = 60,000, AGC target = 3e6, maximum injection time = 50 ms and filter dynamic exclusion: exclusion duration = 30 s; and (2) dd-MS2: isolation window = 4 m/z, resolution = 15,000, AGC target = 5e5, maximum injection time = 80 ms and NCE = 30%. The MS raw data were analyzed and searched by Spectronaut Pulsar X (version 12, Biognosys AG), and a spectral database was established. The standard for library construction was 1% precursor FDR and 1% peptide FDR.

#### DIA-MS analysis

From each fraction, 6 μL was removed and added to 2 μL of 10 × iRT standard peptide, and 2 μL of each sample was separated with nano-LC and analyzed by online electrospray tandem MS. The entire experimental system was an Orbitrap Q Exactive HF mass spectrometer (Thermo Fisher Scientific, MA, USA) connected in series with a Waters Acquity UPLC (Waters, MA, USA) system. Buffer A consisted of 0.1% formic acid aqueous solution, and buffer B consisted of 0.1% formic acid acetonitrile aqueous solution (acetonitrile was 80%). The sample was separated by an analytical column (Thermo Fisher Scientific, MA, USA; Acclaim PepMap C18, 75 μm × 25 cm) at a flow rate of 200 nL/min using the following nonlinear increasing gradient: 0–5 min, 1% B; 5–95 min, 1% B to 28% B; 95–110 min, 28% B to 38% B; 110–115 min, 38% B to 100% B; and 115–120 min, 100% B. The electrospray voltage was 2.0 kV. The MS parameters were set as follows: (1) MS: scan range (m/z) = 350–1250, resolution = 120,000, AGC target = 3e6 and maximum injection time = 20 ms; and (2) DIA: resolution = 30,000, AGC target = 1e6, maximum injection time = auto and NCE = 25.5,27,30. The original MS data and the default parameters of Spectronaut Pulsar X were used to analyze the DIA data. The protein qualitative standard was a precursor threshold of 1.0% FDR. Serum proteins compared between the two specified groups with a threshold of fold change (FC) ≥ 1.50 or ≤ 0.67 and P value ≤ 0.05 were considered as differentially-expressed proteins (DEPs).

### PRM-MS analysis

#### Sample preparation and FASP digestion

The expression of DEPs was preliminarily verified by PRM, which was a target proteomic strategy. For PRM assays, the methods for sample preparation and FASP digestion were the same as previously described for SWATH-MS analysis.

#### MS analysis

The same mass of peptides from each sample was extracted and mixed well, and 2 μg of each sample was separated with nano-LC and analyzed by online electrospray tandem MS. The complete liquid-mass tandem system was composed of a liquid system (Easy nLC system; Thermo Fisher Scientific, MA, USA) and an MS system (Q-Exactive; Thermo Fisher Scientific, MA, USA). Buffer A was composed of 0.1% formic acid aqueous solution, and buffer B was composed of 0.1% formic acid acetonitrile aqueous solution (acetonitrile was 80%). The sample was separated by an analytical column (Thermo Fisher Scientific, MA, USA; Acclaim PepMap RSLC 50 μm × 15 cm, nano viper, P/N164943) at a flow rate of 300 nL/min using the following nonlinear increasing gradient: 0–1 min, 2% B to 8% B; 1–46 min, 8% B to 28% B; 46–56 min, 28% B to 40% B; 56–57 min, 40% B to 90% B; and 57–60 min, 90% B.

The samples were chromatographed and analyzed by a Q Exactive mass spectrometer with the following parameters; analysis time of 60 min; detection method was positive ion; precursor ion scan range of 350–1500 m/z, resolution of the primary MS was 60,000; AGC target was 3e6; and primary maximum IT was 45 ms. The mass-to-charge ratios of peptides and peptide fragments were collected according to the following method: 10 fragment patterns (MS2 scan) were collected after each full scan (MS2 scan); MS2 activation type was HCD; isolation window was 2 m/z; MSMS resolution rate was 15,000, AGC target was 2e5; secondary Maximum IT was 45 ms; and normalized collision energy was 27 eV.

#### PRM precursor ion screening

Proteome Discoverer 2.1 (Thermo Fisher Scientific, MA, USA) software was used to convert the original map files (.raw files) generated by Q Exactive into.mgf files, which were submitted to the MASCOT2.6 server for database retrieval through the built-in tools of the software. The database used was Uniprot_HomoSapiens_20386_20180905. The reliable protein screening criterion was peptide FDR ≤ 0.01.

#### PRM detection

Each sample (2 μg) was separated by nano-LC and analyzed by online electrospray tandem MS. The complete liquid-mass tandem system was composed of a liquid system (Easy nLC system; Thermo Fisher Scientific, MA, USA) and an MS system (Q-Exactive; Thermo Fisher Scientific, MA, USA). Buffer A was composed of 0.1% formic acid aqueous solution, and buffer B was composed of 0.1% formic acid acetonitrile aqueous solution (acetonitrile was 80%). The sample was separated by an analytical column (Thermo Fisher Scientific, MA, USA; Acclaim PepMap RSLC 50 μm × 15 cm, nano viper, P/N164943) at a flow rate of 300 nL/min using the following nonlinear increasing gradient: 0–1 min, 2% B to 8% B; 1–46 min, 8% B to 28% B; 46–56 min, 28% B to 40% B; 56–57 min, 40% B to 90% B; and 57–60 min, 90% B.

The MS parameters were set as follows: (1) Full-MS: scan range (m/z) = 350–1500, resolution = 60,000, AGC target = 1e6 and maximum injection time = 50 ms; and (2) PRM: resolution = 15,000, AGC target = 1e5, maximum injection time = 50 ms, loop count = 14; isolation window = 1.6 m/z and NCE = 27%. Skyline software was used for analysis of PRM data.

### ELISA analysis

The concentrations of PZP (Catalog No. DY8280-05; R&D Systems, MN, USA) and IGFBP3 (Catalog No. DGB300; R&D Systems, MN, USA) in serum were quantified with commercially available ELISA kits according to the manufacturer’s protocol. Most samples were assayed in duplicates, and the average values were reported as pg/mL or ng/mL. The linear correlation between the PRM-MS and ELISA results was calculated using Pearson’s correlation analysis.

### Analysis of public data

The data of *PZP* mRNA expression in the TCGA database was obtained from the Xena website. The correlations between PZP expression and immune cell infiltration were determined by the TIMER database [16]. Besides, the summary of PZP protein was consulted in the HPA database [17, 18].

### Statistical analysis

Statistical analysis was mainly performed in SPSS (v26.0) and GraphPad Prism (v.8.0). Most of the data between the two groups were presented as means ± SDs (Std. Deviations) if not noted and were compared by Student’s t-test or the Mann–Whitney test. Correlation analysis was evaluated by Pearson’s correlation analysis. Receiver-operating characteristic (ROC) analysis was used to assess the specificity and sensitivity of the biomarkers, and the area under the ROC curve (AUC) was estimated for each individual protein. For all analyses, P values less than 0.05 were considered statistically significant.

## Results

### Distribution of tumor location and subtype of lung cancer in T2DM patients

Previous research has indicated that lung cancer is the most common concomitant malignant tumor among patients with diabetes [1]. Thus, to further confirm the distribution of tumor location, we collected clinical information of hospitalized T2DM patients diagnosed with cancers from January 1, 2015, to June 30, 2020. After analyzing the distribution, we found that lung cancer was the highest proportion of malignant tumors (20.84%) followed by digestive tract cancers (colorectum: 12.81%, stomach: 12.32%, and liver: 6.18%) (Table 1). We next analyzed the histological types of T2DM patients with lung cancer. The proportion of histological types was as follows: adenocarcinoma (60.62%), squamous carcinoma (13.86%), small cell carcinoma (3.69%), mixed carcinoma (1.47%), neuroendocrine carcinoma (0.88%), magnocellular carcinoma (0.29%) and other histological types (0.88%) (Table 2). Overall, LAC accounted for the most common tumor in T2DM patients and should be monitored and diagnosed early.

**Table 1:** Distribution of tumor location and gender in patients with T2DM and cancer

Location	Age	Gender				Total	Proportion
		Male		Female			
Lung	66.66 ± 8.36	448	66.08%	230	33.92%	678	20.84%
Colorectum	69.46 ± 10.01	250	59.95%	167	40.05%	417	12.81%
Stomach	68.75 ± 8.83	288	71.82%	113	28.18%	401	12.32%
Liver	67.80 ± 11.26	152	75.62%	49	24.38%	201	6.18%
Prostate	73.79 ± 7.97	193	100.00%	0	0.00%	193	5.93%
Breast	65.97 ± 11.45	4	2.27%	172	97.73%	176	5.41%
Blood	64.80 ± 11.30	97	58.08%	70	41.92%	167	5.13%
Pancreas	68.91 ± 9.24	97	63.82%	55	36.18%	152	4.67%
Bladder	70.02 ± 9.13	103	84.43%	19	15.57%	122	3.75%
Kidney	65.72 ± 10.50	73	66.97%	36	33.03%	109	3.35%
Esophagus	69.38 ± 8.44	67	67.00%	33	33.00%	100	3.07%
Lymphoma	67.11 ± 9.64	53	56.38%	41	43.62%	94	2.89%
Thyroid	53.52 ± 10.75	35	43.21%	46	56.79%	81	2.49%
Gallbladder & biliary	70.13 ± 10.07	40	53.33%	35	46.67%	75	2.30%
Uterus	63.57 ± 12.02	0	0.00%	74	100.00%	74	2.27%
Multiple sites	71.39 ± 10.33	32	69.57%	14	30.43%	46	1.41%
Head and neck	67.00 ± 10.45	24	77.42%	7	22.58%	31	0.95%
Ovary	63.48 ± 8.22	0	0.00%	25	100.00%	25	0.77%
Small intestine	70.86 ± 8.48	12	57.14%	9	42.86%	21	0.65%
Skin	70.88 ± 10.52	10	62.50%	6	37.50%	16	0.49%
Brain	65.13 ± 10.87	8	53.33%	7	46.67%	15	0.46%
Ureter	74.82 ± 6.66	7	63.64%	4	36.36%	11	0.34%
Thymus	58.30 ± 9.09	4	40.00%	6	60.00%	10	0.31%
Other	66.64 ± 10.15	24	61.54%	15	38.46%	39	1.20%
Total	67.70 ± 10.13	2021	62.11%	1233	37.89%	3254	100.00%

**Table 2:** Distribution of histological classification and gender in patients with T2DM and lung cancer

Histological classifications	Age	Gender				Total	Proportion
		Male		Female			
Adenocarcinoma	66.74 ± 8.17	256	62.29%	155	37.71%	411	60.62%
Squamous carcinoma	65.89 ± 8.27	61	64.89%	33	35.11%	94	13.86%
Small cell carcinoma	65.80 ± 7.05	15	60.00%	10	40.00%	25	3.69%
Mixed carcinoma	66.20 ± 6.75	8	80.00%	2	20.00%	10	1.47%
Neuroendocrine carcinoma	67.17 ± 7.49	4	66.67%	2	33.33%	6	0.88%
Magnocellular carcinoma	69.00 ± 5.66	2	100.00%	0	0.00%	2	0.29%
Other	68.67 ± 7.15	6	100.00%	0	0.00%	6	0.88%
Unknown	67.05 ± 9.57	96	77.42%	28	22.58%	124	18.29%
Total	66.66 ± 8.36	448	66.08%	230	33.92%	678	100.00%

### Patient characteristics and study design

Before we screened the potential biomarker that could differentiate LAC in T2DM patients, we first tried to compare the general pathological parameters in the main two groups in the whole set consisting of 40 serum samples from T2DM patients and 40 serum samples from T2DM patients with LAC. In the T2DM group, there were 23 males and 17 females with an average age of 61.05 ± 9.78 years and an average fasting plasma glucose (FPG) of 7.95 ± 1.91 mmol/L. In the T2DM + LAC group, there were 19 males and 21 females with an average age of 64.68 ± 7.10 years and an average FPG of 7.41 ± 2.55 mmol/L. There were no statistically significant differences in sex, age and FPG between the two groups (P > 0.05) (Table 3). Besides, there was also no significant differences in therapeutic regimens for hypoglycemia between these two groups (P > 0.05) (Table 3). Moreover, we compared the concentrations of the most commonly used tumor biomarkers in the clinic between these two groups. The results showed that there were no significant differences in serum AFP (P = 0.101), CEA (P = 0.304), CA125 (P = 0.693) and CA199 (P = 0.994) levels between the T2DM + LAC group and the T2DM group (Table 3). These results suggested that the identification of novel biomarkers is urgently needed for the detection of LAC in T2DM patients.

**Table 3:** Comparison of general information between two groups

General information	T2DM	T2DM + LAC	P value
Gender (male/female)	23/17	19/21	0.371
Hypoglycemic therapy (A/B)^a^	27/13	26/14	0.813
Age (years)	61.05 ± 9.78	64.68 ± 7.10	0.137
AFP (ng/ml)	2.70 ± 1.17	3.21 ± 1.57	0.101
CEA (ng/ml)	2.11 ± 1.09	3.27 ± 3.72	0.304
CA125 (U/ml)	9.50 ± 4.86	12.34 ± 15.66	0.693
CA199 (U/ml)	14.41 ± 10.44	27.51 ± 68.25	0.994
FPG (mmol/L)	7.95 ± 1.91	7.41 ± 2.55	0.287

^a^A, insulin or insulin-dependent therapies; B, insulin-independent therapies or no treatment

Considering the limited values of common tumor biomarkers in T2DM patients, we next performed SWATH-MS, PRM-MS and ELISA analyses to identify and validate novel biomarkers for the detection of LAC in T2DM patients. The overall strategy and simplified workflow are shown in Fig. 1b. Briefly, 20 samples obtained from 5 healthy controls, 5 T2DM patients, 5 LAC patients and 5 T2DM patients with LAC were submitted for SWATH-MS analysis to identify DEPs specific for LAC in patients with T2DM. These results were next validated by PRM-MS and ELISA analysis. Moreover, the validation set consisting of 20 serum samples from T2DM patients and 20 serum samples from T2DM patients with LAC were collected for ELISA analysis and further validation.

### Identification of differentially expressed proteins by SWATH-MS analysis

**Fig. 2:**
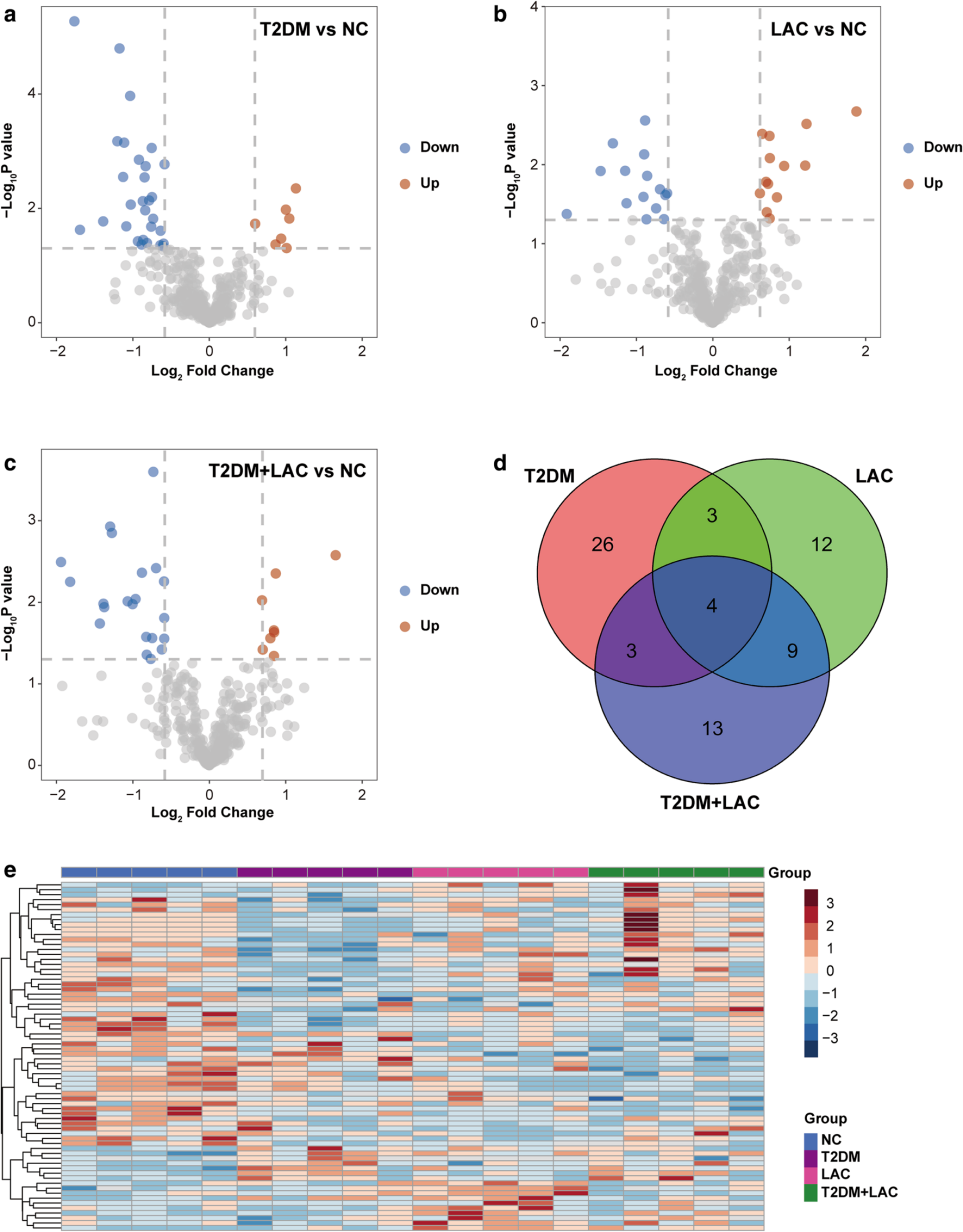
Identification of unique serum proteins in T2DM patients with LAC. **a**–**c** Volcano plot showing the DEPs in the T2DM, LAC and T2DM + LAC patients compared to healthy controls. **d** Venn diagram of the 70 differentially expressed proteins in the serum samples from the four comparisons (T2DM vs. NC, LAC vs. NC and T2DM + LAC vs. NC). In total, 13 proteins were specifically expressed in the T2DM + LAC group. **e** Hierarchical cluster analysis of the 70 DEPs in the NC, T2DM, LAC, and T2DM + LAC serum samples. Note: There were five samples in each group. Pseudocolors indicate differential expression (red, upregulation; blue, downregulation)

Using SWATH-MS analysis, we analyzed global protein changes in serum samples from 20 patients (5 healthy controls, 5 T2DM patients, 5 LAC patients and 5 T2DM + LAC patients). A total of 70 proteins were identified as differentially expressed between these disease groups and the control group (Fig. 2a–c). As shown in Fig. 2d, the three protein lists from the above analysis (T2DM vs. normal, LAC vs. normal and T2DM + LAC vs. normal) were further compared to identify a small group of proteins that were differentially expressed only in the T2DM + LAC group. Overall, 13 proteins were found to be unique in patients with T2DM + LAC (Fig. 2d). Among these proteins, 7 candidates exhibited differential expression between the T2DM + LAC and T2DM groups, including 2 upregulated proteins and 5 downregulated proteins (Tables 4 and 5). To arrange the samples according to similarities in protein expression patterns, we performed a hierarchical cluster analysis of the 70 DEPs as previously described [19]. Cluster analysis indicated a clear separation of the four groups (Fig. 2e).

**Table 4:** List of serum differential proteins identified by SWATH-MS in patients with T2DM + LAC and T2DM

Protein	Accession	Protein description
ADIPO	Q15848	Adiponectin
CCD87	Q9NVE4	Coiled-coil domain-containing protein 87
CXCL7	P02775	Platelet basic protein
FBLN1	P23142	Fibulin-1
FHR1	Q03591	Complement factor H-related protein 1
FRPD2	Q68DX3	FERM and PDZ domain-containing protein 2
HBB	P68871	Hemoglobin subunit beta
HV145	A0A0A0MS14	Immunoglobulin heavy variable 1–45
IGFBP3	P17936	Insulin-like growth factor-binding protein 3
LV316	A0A075B6K0	Immunoglobulin lambda variable 3–16
PZP	P20742	Pregnancy zone protein
SRGN	P10124	Serglycin
ZN350	Q9GZX5	Zinc finger protein 350

**Table 5:** Unpaired T-test of the quantitative proteomic results for protein expression in the T2DM + LAC group in comparison with the control and T2DM groups

Protein	T2DM + LAC vs. control		T2DM + LAC vs. T2DM	
	Fold change	P value	Fold change	P value
ADIPO	−1.873	0.004	−1.629	0.199
CCD87	−1.664	< 0.001	−1.440	< 0.001
CXCL7	−2.700	0.018	−2.245	0.115
FBLN1	−1.506	0.016	−1.372	0.098
FHR1	1.613	0.010	1.510	0.025
FRPD2	−2.097	0.010	−1.751	0.035
HBB	−3.534	0.006	−2.188	0.019
HV145	−1.702	0.049	−1.392	0.138
IGFBP3	−1.765	0.044	−2.371	0.003
PZP	1.801	0.023	2.488	0.022
SRGN	1.624	0.038	1.573	0.131
ZN350	−1.679	0.028	−1.810	0.006

LV316 was excluded because of be missing in most samples

### Verification of selected candidate proteins by PRM-MS ELISA analyses

**Fig. 3:**
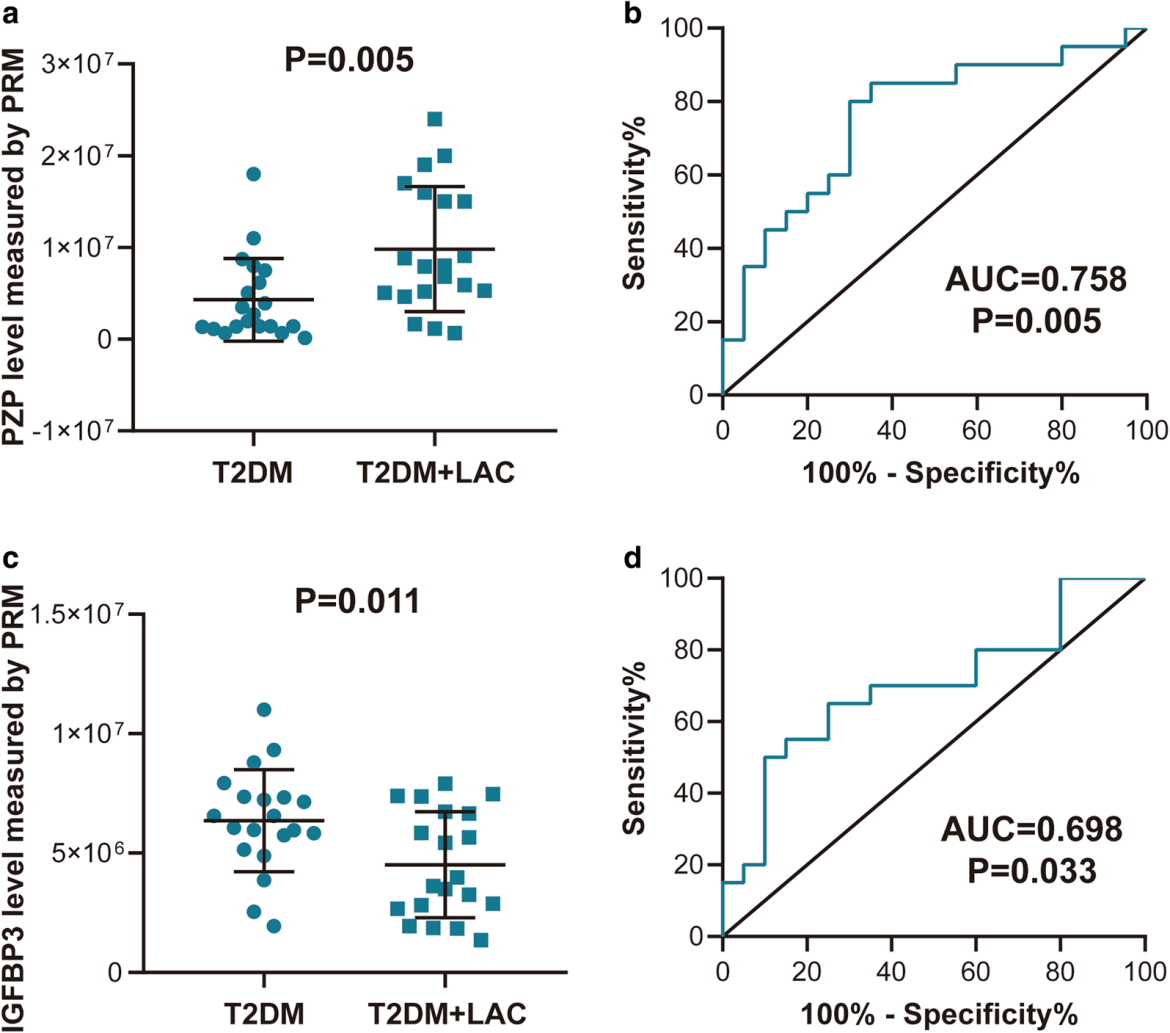
Validation of selected candidate proteins by PRM-MS analysis. Differential expression of (**a**) PZP and (**c**) IGFBP3 in the T2DM + LAC and T2DM groups. ROC analysis of the diagnostic value of (**b**) PZP and (**d**) IGFBP3 for the detection of LAC in T2DM patients

Of the 13 proteins identified as DEPs in patients with T2DM + LAC by SWATH-MS analysis, 7 proteins showed significant dysregulation between T2DM + LAC and T2DM, including CCD87, FHR1, FRPD2, HBB, IGFBP3, PZP, and ZN350 (Table 5). We next used targeted PRM-MS to provide high sensitivity relative peptide quantification for validation. A total of 4 proteins were detected by PRM-MS, and significant differential expression of 2 of these candidate proteins was confirmed, namely, PZP and IGFBP3 (Fig. 3a–d, Additional file 1: Figure S1).

**Fig. 4:**
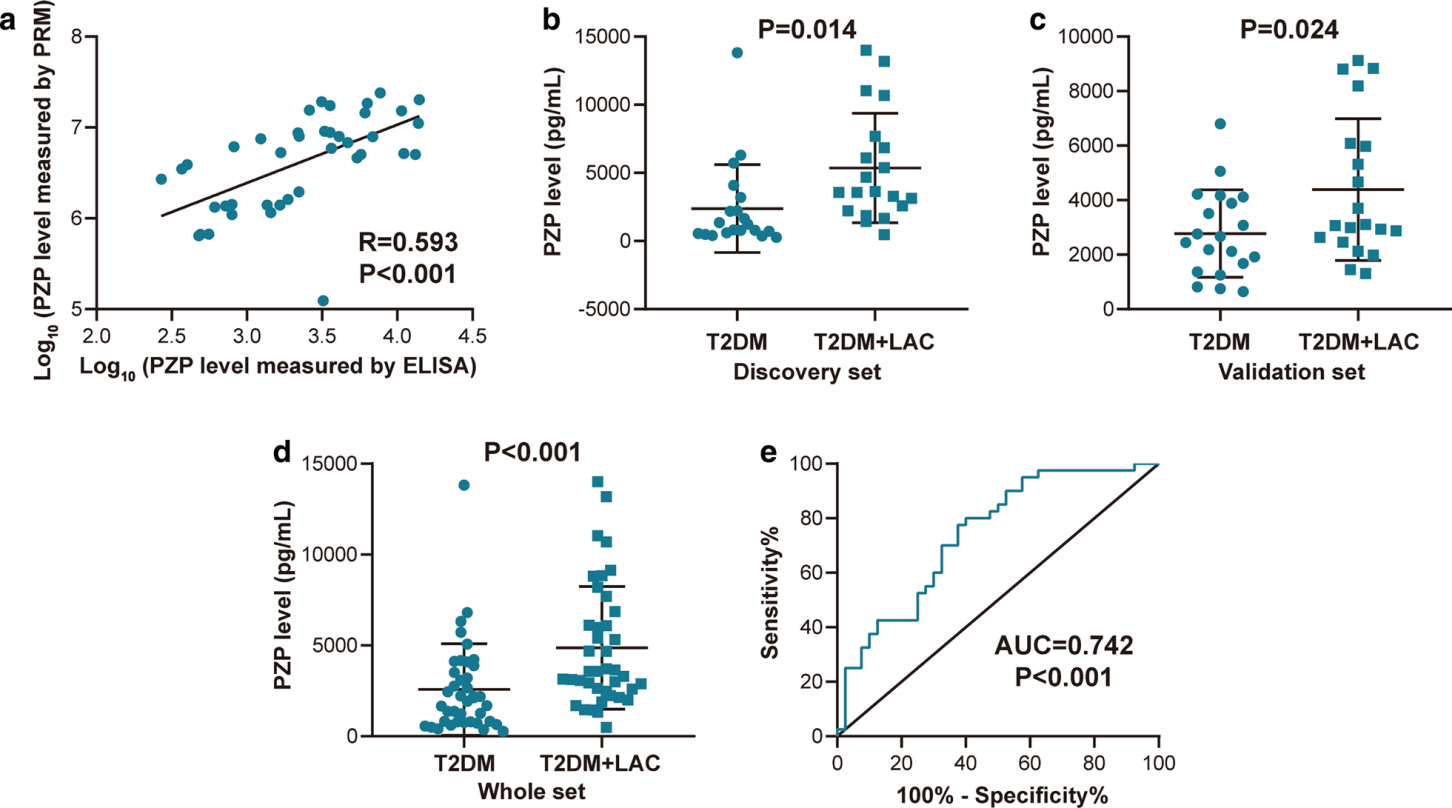
Validation of selected candidate proteins by ELISA analysis. **a** Correlation between PRM-MS and ELISA assay results for PZP. **b** Differential expression of PZP in the T2DM + LAC and T2DM groups in the discovery set. **c** Differential expression of PZP in the T2DM + LAC and T2DM groups in the validation set. **d** Differential expression of PZP in the T2DM + LAC and T2DM groups in the whole set. **e** ROC analysis of the diagnostic value of PZP for the detection of LAC in T2DM patients in the whole set

We next validated the protein abundance changes of PZP and IGFBP3 using commercially available antibodies and ELISA kits. The concentration-dependent standard curve is shown in Additional file 2: Figure S2. To evaluate the feasibility of developing an assay that could be more easily deployed in a clinical environment, we assessed the transferability of the PRM-MS-based results to ELISA. The levels of PZP and IGFBP3 were quantified by commercially available ELISA kits, and the correlation with the results obtained by PRM-MS was evaluated. The results showed a linear correlation for PZP but not IGFBP3 (Fig. 4a, Additional file 3: Figure S3). In addition, the level of PZP between the T2DM + LAC and T2DM groups was significantly different in the discovery set, the validation set and the whole set, and the ROC analysis indicated an AUC of 0.742 (Fig. 4b–e). However, no significant difference was observed in IGFBP3 levels between these two groups (Additional file 3: Figure S3). In summary, detection of PZP level provides enough sensitivity and specificity, and it merits further validation in larger cohort samples.

## Discussion

As two common chronic non-communicable diseases, more and more studies have realized the correlation between lung cancer and T2DM. In a meta-analysis, Lee et al*.* systematically analyzed 34 observational studies and found that after adjusting for smoking and other variables, T2DM was an independent risk factor for the occurrence of lung cancer with a relative risk of 1.11 and a 95% CI of 1.02 to 1.20 [20]. At the same time, T2DM is also related to the risk of lung cancer death. Tseng et al*.* conducted a prospective study of 244,920 T2DM patients with a 12-year follow-up and found that the LC mortality rate of T2DM patients was significantly higher [21]. In the present research, we systematically analyzed the distribution of tumor location and subtype of lung cancer in T2DM patients. The results revealed that lung cancer was the most common malignant tumor in patients with T2DM, with LAC accounting for the majority of cases. Moreover, unlike pancreatic cancer, which has the highest increased risk in patients with T2DM, the early diagnosis and treatment of lung cancer can significantly improve prognosis [22, 23]. Therefore, more strategies for the early screening of LAC in T2DM patients should be further explored.

Although cytology is the gold standard for the diagnosis of malignancies, serum biomarkers are also invaluable in the screening and auxiliary diagnosis of malignant tumors as well as monitoring curative effects [24, 25]. The serum proteome holds significant interest as a potential source of biomarkers and is an easily accessible fluid for auxiliary diagnosis. Four tumor biomarkers, including AFP, CEA, CA125 and CA199, are widely used in clinical practice. An observational study presented by Chen et al*.* revealed the association between the levels of these biomarkers and the tumor stage of LAC. Serum AFP was not correlated with T stage, N stage or M stage, but serum CEA and serum CA125 were positively correlated with T stage, N stage and M stage. Serum CA199 was not correlated with T stage but was positively correlated with N stage and M stage [26]. However, it is unknown whether these four biomarkers help to identify LAC in patients with T2DM. In our study, the results indicated that there were no significant differences in serum CEA, AFP, CA125 and CA199 levels between the T2DM + LAC group and the T2DM group, indicating an urgent need for the identification of promising biomarkers for the detection of LAC in T2DM patients.

The MS-dependent identification of serum biomarkers has recently emerged [27, 28]. SWATH-MS is a newly developed technology, which combines the advantages and characteristics of traditional “shotgun” proteomics and selective reaction monitoring/multiple reaction monitoring (SRM/MRM) [12]. SWATH-MS technology can obtain all fragment information of all ions in the sample without omission and difference, while PRM technology can achieve the absolute quantification of protein expression. The combination of the two strategies can be used for the efficient, comprehensive and accurate screening of potential biomarkers [29, 30]. In this study, we performed SWATH-MS analysis to identify DEPs specific for LAC in patients with T2DM, and these potential biomarkers were validated by PRM-MS and ELISA analysis in the discovery and validation cohort.

To identify a small group of proteins that were differentially expressed in the T2DM + LAC group, we compared the three protein lists (T2DM + LAC vs. normal, T2DM vs. normal and LAC vs. normal) and identified 13 proteins that were unique in patients with T2DM + LAC. Among these proteins, 7 candidates exhibited differential expression between the T2DM + LAC and T2DM groups. To identify useful diagnostic indicators from these 7 proteins, we conducted further validation by PRM-MS. The results showed that 4 proteins were detected by PRM-MS and that significant differential expression of 2 of these candidate proteins was confirmed, namely, PZP and IGFBP3. As a first step toward clinical implementation, the diagnostic biomarker was assessed by ELISA. Immunoassays continue to be the preferred method for clinical validation and further application in clinical practice [31]. The PZP levels were significantly different between the T2DM + LAC and T2DM groups, and the ROC analysis indicated an AUC of 0.742 in the whole set. However, no significant difference in IGFBP3 levels was observed between these two groups.

PZP is associated with pregnancy, and it is produced in the liver, placenta and other tissues. The blood concentration of PZP increases during pregnancy [32]. Mechanically, elevated estrogen levels during pregnancy may regulate PZP levels [33]. Moreover, elevated PZP has been identified as an indicator associated with *P. aeruginosa* infection. Sputum but not serum concentrations of PZP have been significantly associated with the Bronchiectasis Severity Index, the frequency of exacerbations and symptoms [34]. Previous research has also uncovered the role of PZP in cancers. In hepatocellular carcinoma, PZP has low expression in tumor tissues, and the downregulation of PZP is correlated with poor clinical outcomes [35]. Our research identified and validated PZP as a novel serum biomarker for screening LAC in patients with T2DM by SWATH-MS, PRM-MS and ELISA analyses. Besides, we also analyzed the expression of PZP and its correlations with immune cell infiltration in lung cancer. The results showed that *PZP* mRNA was downregulated in lung cancer tissues and significantly correlated with immune cell infiltration (Additional file 4: Figure S4A-B). However, in the TCGA database, not all patients have T2DM before the diagnosis of lung cancer. Besides, the TCGA database only provides gene expression data at the mRNA level. Serum biomarkers are not only derived from tumor cell, but may also be released by tumor-related immune cells [36]. According to the HPA database, PZP is highly expressed in immune cells, including T cells and macrophages. In previous research, *P. aeruginosa* infection-induced PZP elevation was derived from neutrophils [34]. Therefore, serum PZP may be derived from tumor-related immune cells, but further studies still need to confirm the source of PZP and its diagnostic value by large-scale analysis.

## Conclusion

In conclusion, the present results revealed that PZP could be used as a novel serum biomarker for the detection of LAC in T2DM patients, which will enhance auxiliary diagnosis at an early stage. However, the present study was conducted using a small sample size at a single center. Hence, the performance of the biomarker panel needs to be validated in a prospective, multicentric study with a higher number of patients.

## Supplementary Information

**Additional file 1: Figure S1**. Validation of selected candidate proteins by PRM-MS analysis. Differential expression of (A) HBB and (B) CFHR1 in the T2DM+LAC and T2DM groups.

**Additional file 2: Figure S2. **Standard curve of ELISA assay for (A) PZP and (B) IGFBP3.

**Additional file 3**: **Figure S3. **Validation of IGFBP3 by ELISA analysis. (A) Correlation between PRM-MS and ELISA assay results for IGFBP3. (B) Differential expression of IGFBP3 in the T2DM+LAC and T2DM groups.

**Additional file 4: Figure S4.** (A) The expression of PZP mRNA in lung cancer and (B) its correlations with immune cells infiltration.

## Data Availability

The data used to support the findings of this study are available from the corresponding author upon request.

## Abbreviations

T1DM: Type 1 diabetes mellitus
T2DM: Type 2 diabetes mellitus
IGF: Insulin-like growth factor
MS: Mass spectrum
SWATH-MS: Sequential windowed acquisition of all theoretical fragment ion mass spectrum
DIA: Data-independent acquisition
PRM-MS: Parallel reaction monitoring MS
LAC: Lung adenocarcinoma
IGFBP3: Insulin-like growth factor binding protein 3
T2DM + LAC: T2DM patients with LAC at TNM stage 1
FC: Fold change
DEPs: Differentially expressed proteins
ROC: Receiver-operating characteristic
AUC: The area under the ROC curve
FPG: Fasting plasma glucose
SRM/MRM: Selective reaction monitoring/multiple reaction monitoring
