# Supplementary material for: Discovery and validation of PZP as a novel serum biomarker for screening lung adenocarcinoma in type 2 diabetes mellitus patients
Source: Cancer Cell Int. 2021 Mar 10;21:162. doi: 10.1186/s12935-021-01861-8 (PMC7945354; doi:10.1186/s12935-021-01861-8)
Supplement: Supplementary file 1 — Additional file 1: Figure S1. Validation of selected candidate proteins by PRM-MS analysis. Differential expression of (A) HBB and (B) CFHR1 in the T2DM+LAC and T2DM groups. [file 12935_2021_1861_MOESM1_ESM.docx]

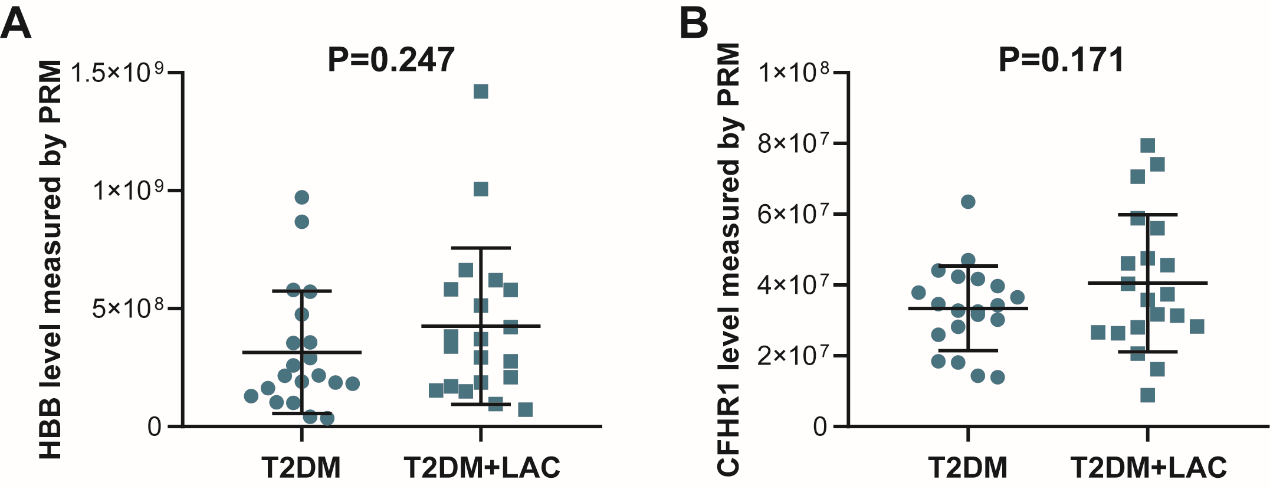


**Figure S1. Validation of selected candidate proteins by PRM-MS analysis.**

Differential expression of (A) HBB and (B) CFHR1 in the T2DM+LAC and T2DM groups.
