# Supplementary figures and images for: Discovery and validation of PZP as a novel serum biomarker for screening lung adenocarcinoma in type 2 diabetes mellitus patients
Source: Cancer Cell Int. 2021 Mar 10;21:162. doi: 10.1186/s12935-021-01861-8 (PMC7945354; doi:10.1186/s12935-021-01861-8)

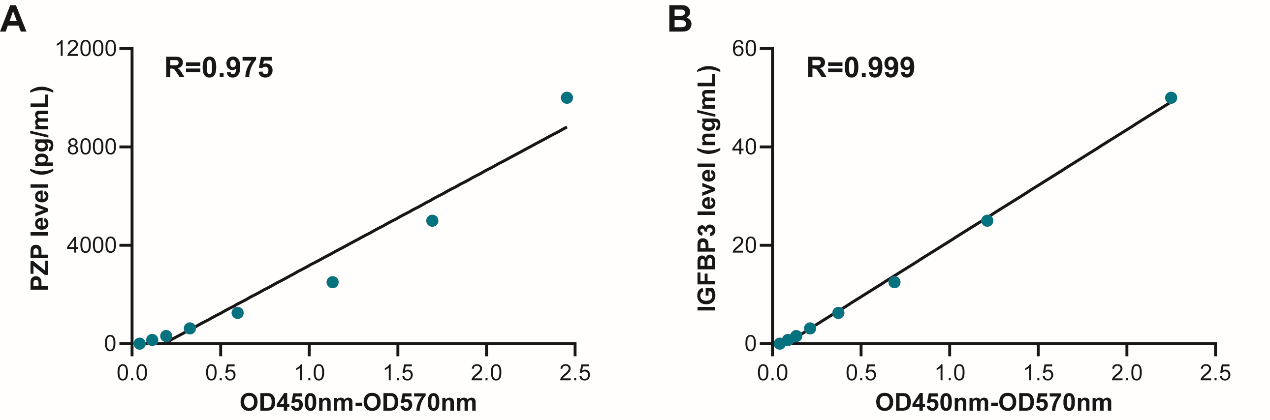


**Figure S2. Standard curve of ELISA assay for (A) PZP and (B) IGFBP3.**

Supplement: Supplementary file 2 — Additional file 2: Figure S2. Standard curve of ELISA assay for (A) PZP and (B) IGFBP3. [file 12935_2021_1861_MOESM2_ESM.docx]
