# Supplementary material for: Discovery and validation of PZP as a novel serum biomarker for screening lung adenocarcinoma in type 2 diabetes mellitus patients
Source: Cancer Cell Int. 2021 Mar 10;21:162. doi: 10.1186/s12935-021-01861-8 (PMC7945354; doi:10.1186/s12935-021-01861-8)
Supplement: Supplementary file 3 — Additional file 3: Figure S3. Validation of IGFBP3 by ELISA analysis. (A) Correlation between PRM-MS and ELISA assay results for IGFBP3. (B) Differential expression of IGFBP3 in the T2DM+LAC and T2DM groups. [file 12935_2021_1861_MOESM3_ESM.docx]

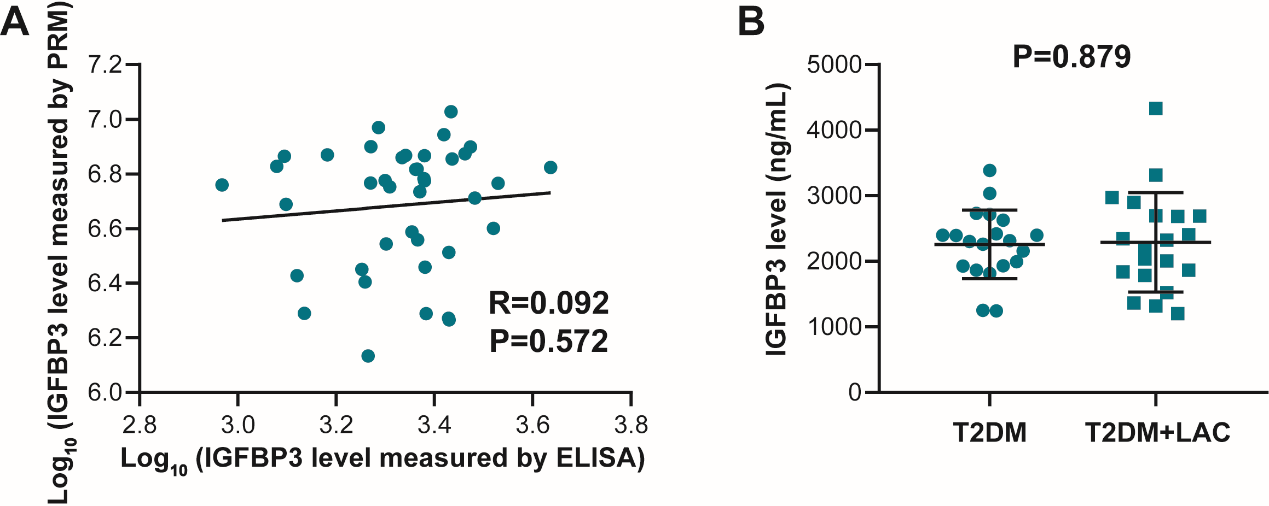


**Figure S3. Validation of IGFBP3 by ELISA analysis**

(A) Correlation between PRM-MS and ELISA assay results for IGFBP3. (B) Differential expression of IGFBP3 in the T2DM+LAC and T2DM groups.
