# Supplementary material for: Discovery and validation of PZP as a novel serum biomarker for screening lung adenocarcinoma in type 2 diabetes mellitus patients
Source: Cancer Cell Int. 2021 Mar 10;21:162. doi: 10.1186/s12935-021-01861-8 (PMC7945354; doi:10.1186/s12935-021-01861-8)
Supplement: Supplementary file 4 — Additional file 4: Figure S4. (A) The expression of PZP mRNA in lung cancer and (B) its correlations with immune cells infiltration. [file 12935_2021_1861_MOESM4_ESM.docx]

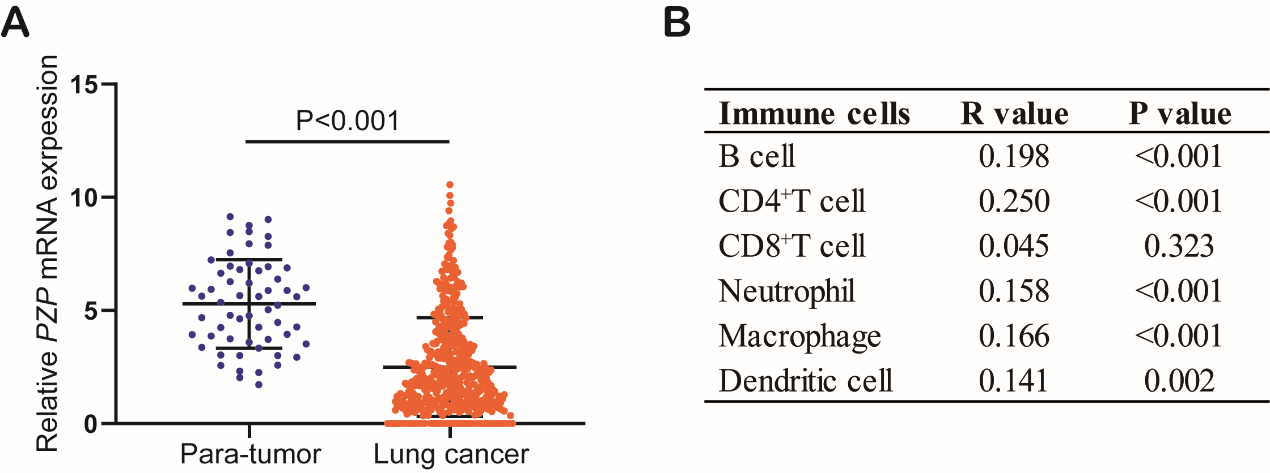


**Figure S4.** (A) The expression of PZP mRNA in lung cancer and (B) its correlations with immune cells infiltration.
